# Biomechanical cadaveric comparison of patellar ligament suture protected by a steel cable versus a synthetic cable

**DOI:** 10.1186/s40634-017-0084-6

**Published:** 2017-03-23

**Authors:** P. Bouget, C. Breque, J. S. Beranger, J. P. Faure, F. Khiami, T. Vendeuvre

**Affiliations:** 1 Department of Orthopedics and Traumatology, University Hospital Center of Poitiers, Poitiers, France; 2 ABS Lab, University School of Medicine of Poitiers, Poitiers, France; 3 Department of Orthopedics and Traumatology, André Mignot Hospital, Hospital Center of Versailles, Versailles, France; 4 Department of Visceral Surgery, University Hospital Center of Poitiers, Poitiers, France; 5 Department of Orthopedics and Traumatology, La Pitié Salpêtrière Hospital, Public Hospital of Paris, Paris, France

**Keywords:** *Patellar ligament*, *Patellar tendon*, *Frame*, *Rupture*, *Terylene*, *Polyester*

## Abstract

**Background:**

Purpose and hypothesis: Patellar ligament rupture is a rare disabling pathology requiring a surgical ligament suture protected by a frame. The gold standard is the steel cable, but its rigidity and the necessity of a surgical re-intervention for its removal render it unsatisfactory. The objective of this paper is to quantify the mechanical protection provided by the terylene® in comparison with steel.

**Methods:**

Twenty-four knees of 12 fresh frozen cadaveric subjects were divided into 2 homogeneous groups (terylene and steel) of 12 knees (mean age = 69.3 years). Proximal ligament repair was performed according to a three-tunnel transosseous reinsertion technique. Mechanical tests were performed in flexion to simulate movement of the knee. The interligament gap and the amplitude angulation of the knee were measured by a system of extensometer and optical goniometer. Mechanical analysis permitted calculation of flexion amplitude for a ligament gap of 1 and 2 mm taking as initial angle the adjusting angle of pretension of the protection frame. Study of deformations of frames was performed. Statistical analysis was performed with a Wilcoxon Mann Whitney test.

**Results:**

There is no significant difference in protection of the ligament suture between the “terylene” and “steel” groups. Mean flexion amplitudes (mΔF) show no significant differences between the 2 groups for a distension of the suture of 1 mm (m ΔF terylene1 = 4.74 °; mΔF steel1 = 5.91°; *p* = 0.198) and 2 mm (mΔF terylene2 = 8.71°; mΔF steel2 = 10.41°; *p* = 0.114). Elastic deformation of terylene was significantly greater than that of steel (*p* = 0.0004).

**Conclusion:**

Suture protection of the patellar ligament by a terylene wire is not significantly different from that provided by steel frame. The elastic properties of terylene and absence of a need for re intervention to secure its removal lead us towards its use in acute ruptures of the patellar ligament. The main limits involve the properties of the chain extenders with no contraction/muscle shortening and partial dehydration of tendons and ligaments and the mean age of 69.3 years.

Level 5.

**Electronic supplementary material:**

The online version of this article (doi:10.1186/s40634-017-0084-6) contains supplementary material, which is available to authorized users.

## Background

Patellar ligament rupture is a rare pathology (Badelon et al. 1985; Coudane & Huttin 1999; Otsubo et al. 2017; Saragaglia et al. 2013) that compromises knee function. It is mostly complete and mainly affects the tip of the patella (43%) (Ait Si Selmi et al. 1999). Acute ruptures occur in young (less than 40 years) (Boggione & Marmorat 2004; Siwek & Rao 1981), male (Clayton & Court-Brown 2008; Shelbourne et al. 2001) and athletic subjects with chronic tendinopathy-related strain injuries (Roudet et al. 2015) (56% sports accidents in Roudet et Al. series (2015)).

Treatment is always surgical (Ait Si Selmi et al. 1999) by patellar ligament suture. Protection by a frame is a necessity (Shelbourne et al. 2001), permitting early mobilization to limit knee stiffness and quadricipital adherences and amyotrophy (Lindy et al. 1995) and to give rise to better functional recovery.

The gold standard is a metallic cable as described by the A.O Foundation (Patella 34-A1 ORIF. https://www2.aofoundation.org. Accessed 12 Mar 2008) and used in several studies (Bhargava et al. 2004; Kasten et al. 2001; Ramseier et al. 2006; Roudet et al. 2015). According to our experience, this frame is unsatisfactory because of its rigidity, its propensity to break, the need for reintervention to remove the material, as well as its tendency to turn the patella in the sagittal plane, or even descend it (Ait Si Selmi et al. 1999).

While some clinical studies have used in vivo synthetic frames (Kasten et al. 2001; Lindy et al. 1995; Miskew et al. 1980), no biomechanical study has compared metal and synthetic frames. The terylene wire used in this study is a braided non absorbable polyester which has been used for several years with very good biocompatibility (Li et al. 2006; Quester et al. 2002) and a conservation of its biomechanical properties (Privalova et al. 1988) in vivo.

The main objective of this paper was to compare the protection of the ligament suture provided by a standard metallic frame and the protection provided by a synthetic frame in terylene, permitting an efficient flexible setting and that would not require further intervention for the metalwork removal. Secondarily, the distension properties of different frames by tracking markers were analyzed.

## Methods

### Study design and groups

This biomechanical experimental study was performed at the ABS lab of the faculty of Medicine and Pharmacy of the University of Poitiers in June 2015. Twelve knees on 6 fresh frozen anatomic male subjects and 12 knees on 6 fresh frozen anatomic female subjects were removed with governmental authorization (accreditation number is DC-2008-137).

Exclusion criteria were history of knee surgery, particularly compromising the integrity of the extensor mechanism and a history of patellar ligament rupture or of patella fracture and a poor bone quality. Groups were comparable by age (mean G_steel_ = 69.3 years [53.69; 84.97]; mean G_terylene_ =69.3 years [53.69; 84.97], sex (sex G_steel_ =6 m/6f; sex G_terylene_ =6 m/6f) and by laterality of knees (laterality G_steel_ =6 L/6R; laterality G_terylene_ =6 L/6R) by distribution of a knee of the same subject in each group to obtain two groups of 12 knees: a group protected by a steel frame (G_steel_) and the other by a terylene frame (G_terylene_) (Additional file 1).

Specimens consisted of the patella, the patellar ligament and the proximal part of the tibia up to 10 cm below the anterior tibial tuberosity.

### Preparation of specimens for measurements

After thawing and hydration of the specimens by immersing them in a saline solution at 20 °C for 15 min, a complete section of proximal patellar ligament was performed. Patellar ligament was reinserted into a patellar trench by transosseous suture with a non resorbable mersuture™ size 3 (*Ethicon*; ref F1513) (Clayton & Court-Brown 2008).

Tibial metaphysis was impacted in the tibial part of a hinge prosthesis.

The position of the patella was settled respecting the Caton-Deschamps index (Caton 1989) equal to 1, and was maintained by a fixed metallic hook in order to overcome sliding movement of the quadriceps.

The frames were set up passing transversely through the patella and the tibia at the level of the anterior tibial tuberosity independently of their structures. Steel frames were produced with steel wire size 6 (*Péters surgical*; ref 31761) and terylene frames with terylene wire size 7 (*Péters surgical*; ref 22020). They were tensioned and tied as soon as the reinsertion was estimated under acceptable pressure, that is to say borderline the reopening of the suture, and acceptable tension was ensured by bending the hinge prosthesis. Finally, the distal portion of the tibial pin was placed in the jaws of the Tinius Olsen machine and the patellar hook fixed to the femoral pin.

### Mechanical tests

**Fig. 1:**
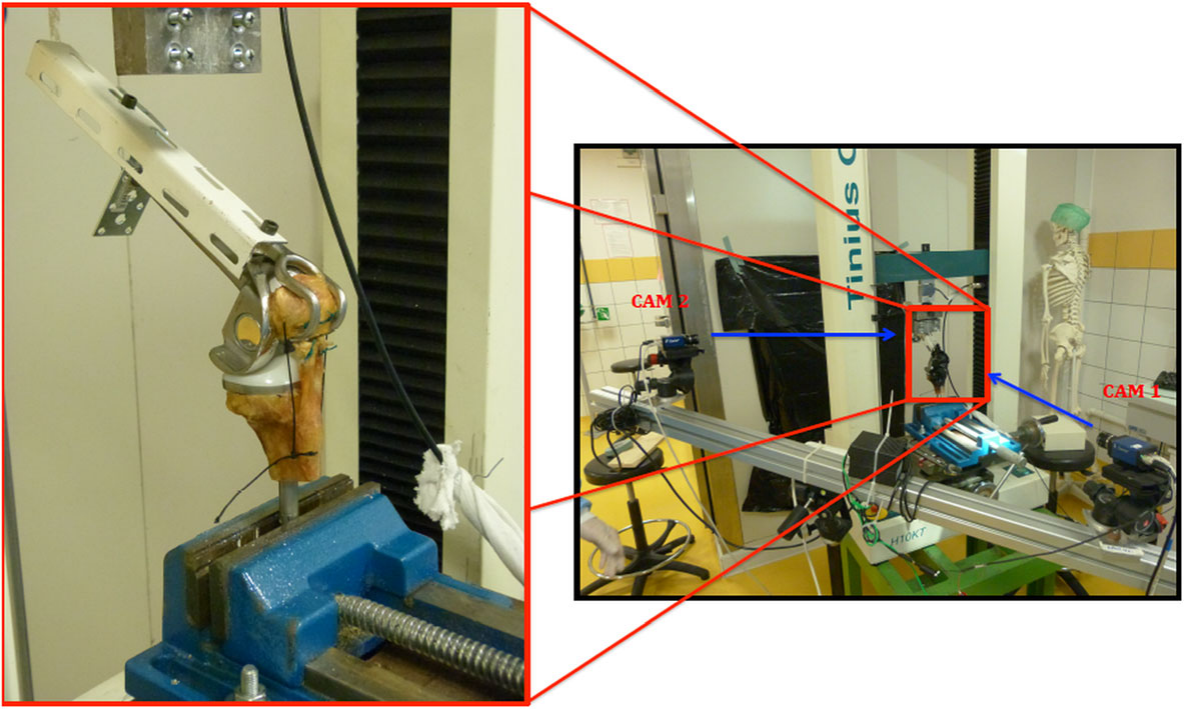
*Specimens in the mechanical stress machine Tinius Olsen: (Camera 1 optical extensometer: measures the inter-tendon gap of the suture; Camera 2 optical goniometer: measured angulation amplitude of the knee)*

**Fig. 2:**
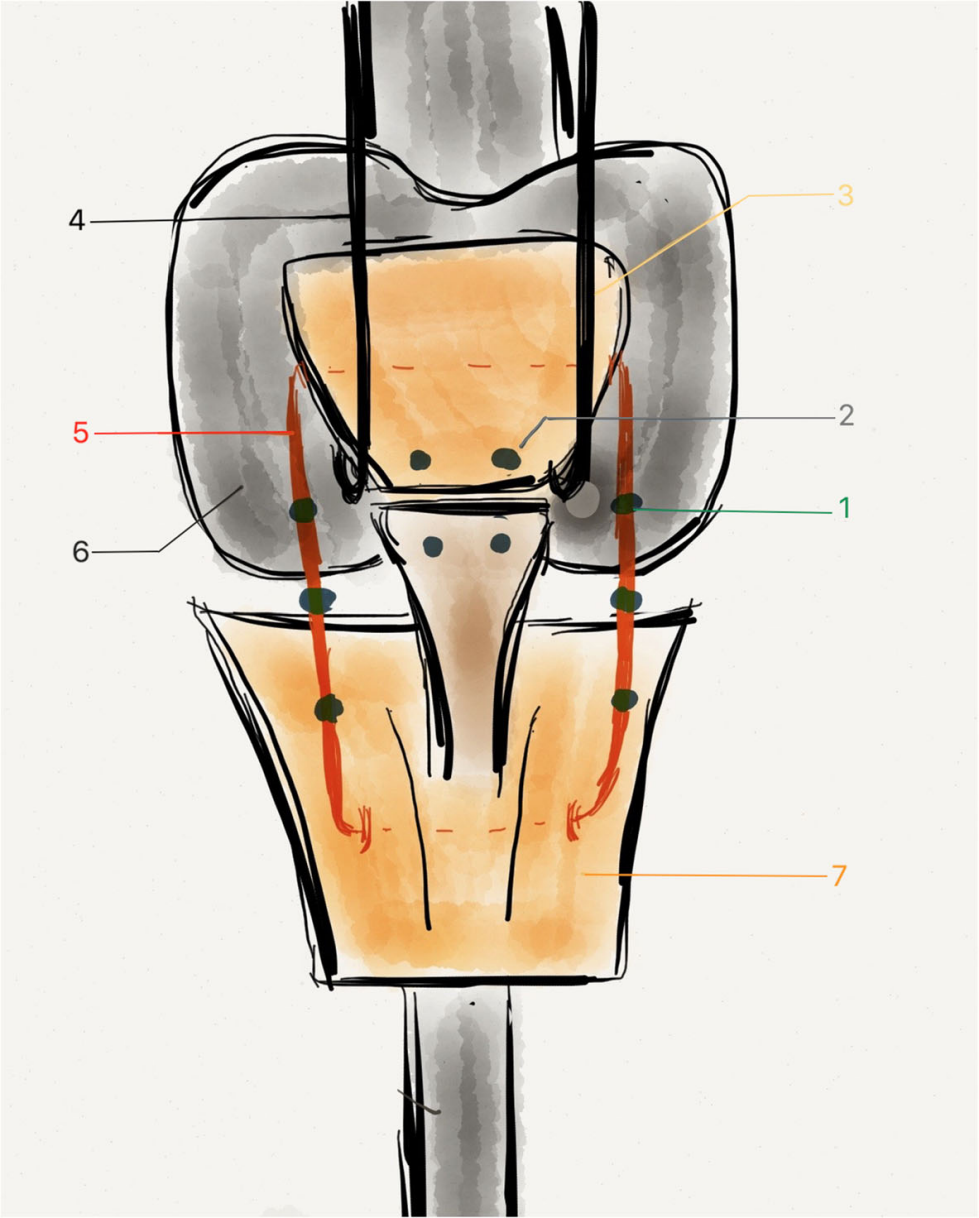
*Markers and front view: (1: marker/pin positioned on the frame, 2: marker/pin positioned on either side of the ligament suture, 3 Patella, 4: patellar bracket, 5: protection frame, 6: Femoral component, 7: Tibial Metaphyso-epiphyseal portion)*

They consisted in the realization of a morphological knee flexion, using a Tinius Olsen 10 kN (Tinius Olsen Machine Compagny; England) compression machine (Fig. 1). In these tests, three mechanical parameters were measured: the amplitude angulation of the knee, inter- ligament gap and lengthening of frame. Angulation amplitude of the knee was measured by an optical goniometer (Fig. 1, camera 1) (Camera Genie, Teledyne-Dalsa®) by positioning markers on the keel of the Link hinge prosthesis to characterize knee posture. The inter-tendon gap of the suture was measured using an optical extensometer (Fig. 1, camera 2) (camera Genie, Teledyne-Dalsa®) by positioning markers on either side of the suture at a distance of 4 mm. With the same optical extensometer, lengthening of lateral bracing of the frame was also measured with markers positioned on the lateral braces of the frame (Fig. 2). Positioning of these different markers was automatically performed by the technique of marker tracking (Deftac ® software by Peprime lab, Poitiers, France).

**Fig. 3:**
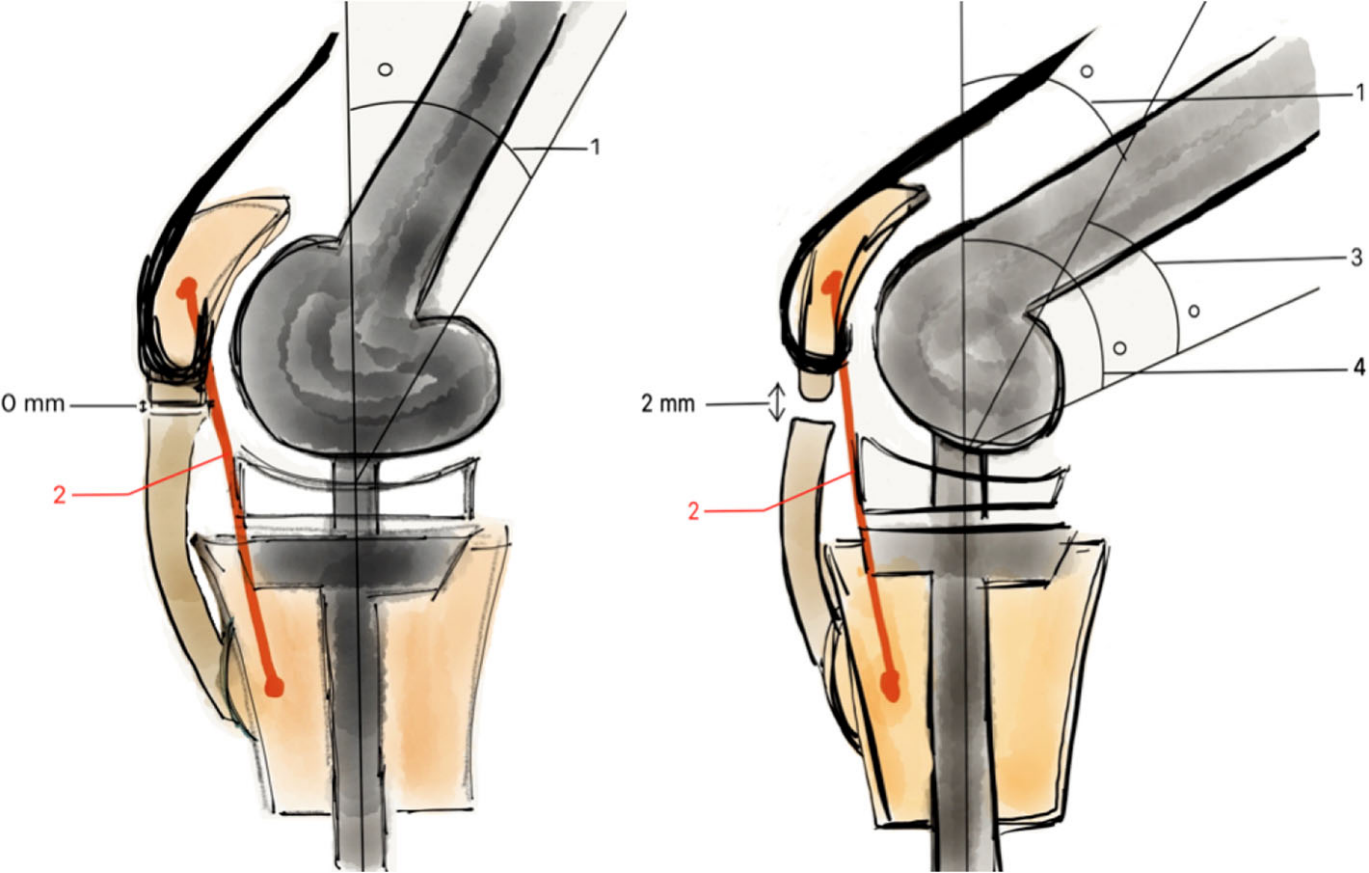
*Side-view of the experimental set-up: With flexion of pretension of the frame (left) and flexion for an inter-suture gap of 2 mm (right). – (1 : Flexion angle (°) of pretension of the frame with inter-suture gap = 0 mm, 2 : Protection frame, 3 : Delta flexion (°) for inter-suture gap of 2 mm (ΔF 2 mm), 4 : Total flexion (°) for inter-suture gap of 2 mm)*

This was used to calculate flexion amplitudes for a gap of the ligament suture of 1 and 2 mm, taking as initial angle the seat angle and knotting of the frame, which is the flexion associated with initiation of the disunion of ligament suture. Flexion amplitude was called delta flexion angle (ΔF). Example: ΔF 2 mm = knee flexion angle for a distension of the suture of 2 mm—flexion angle and initial knotting of the frame (Fig. 3). The percentage of deformation of the frames (% def) for a suture deviation of 1 and 2 mm were calculated too. Example: % def 2 mm = (distance between the two markers of the frame at flexion angle and initial knotting of the frame * 100)/distance between the two markers of the frame for distension of the suture of 2 mm.

### Statistical analysis

Statistical analysis was performed using Mann Whitney Wilcoxon tests on the R software 2.15 (R Foundation for Statistical Computing). Statistical significance was set at *p* < 0,05.

## Results

Mean delta flexion angles (m ΔF) were for 1 mm of distension of the suture: m ΔF terylene1 = 4.74 ° [2.01°; 7.39°]; m ΔF steel1 = 5.91° [0.07°; 11.75°]; and were for 2 mm: m ΔF terylene2 = 8.71° [4.59°; 12.9°]; m ΔF steel2 = 10.41° [0.79°; 20.03°].

There was no significant difference between the “terylene frame” and “steel frame” groups in delta flexion angles for a distension of the suture of 1 mm (*p* = 0.198) and 2 mm (*p* = 0.114) (Table 1) (Additional file 2). There was therefore no significant difference (*p* > 0.05) between the two groups as regards protection of ligament suture.

**Table 1:** Mean delta flexion angles (m ΔF) for inter-suture gap of 1 and 2 mm

	1 mm	2 mm
m ΔF terylene	4.74 ° [2.01°; 7.39°]	8.71° [4.59°; 12.9°]
m ΔF steel	5.91° [0.07°; 11.75°]	10.41° [0.79°; 20.03°]
Mann Whitney Wilcoxon test (*p*)	0.198	0.114

*[] = confidence interval of 95% for inter-suture gap of 1 and 2 mm*

Deformation of the different frames was measured as a percentage of deformation during flexion in comparison with its initial state of pre-tension. The average deformation percentages (% def) of terylene and steel were for 1 mm of distension of the suture: %def terylene1 = 1.62 [0.09; 3.15]; %def steel 1 =−1.61 [−3.52; 0.28]; and for 2 mm of distension of the suture: %def terylene2 = 2.63 [0.61; 4.65]; %def steel 2 =−2.73 [−6.32; 0.86].

A significant difference was found between terylene frames and steel frames in their deformation percentages for a distension of the suture of 1 mm (*p* = 0.0004) and 2 mm (*p* = 0.0004) (Table 2) (Additional file 2). Terylene frame was therefore significantly more deformable than the steel frame.

**Table 2:** Mean deformation percentage (%def) of frames for inter-suture gaps of 1 and 2 mm

	1 mm	2 mm
%def terylene	1.62 [0.09; 3.15]	2.63 [0.61; 4.65]
%def steel	−1.61 [−3.52; 0.28]	−2.73 [−6.32; 0.86]
Mann Whitney Wilcoxon test (*p*)	0.0004	0.0004

*[] = Confidence interval of 95% [] for inter-suture gap of 1 and 2 mm*

After mechanical tests, specimens were physically examined to identify failure mechanisms. It was noticed that lengthening of terylene was due not only to its elasticity but also to a retightening of its knot and then a slipping on 6 of the 12 specimens.

**Fig. 4:**
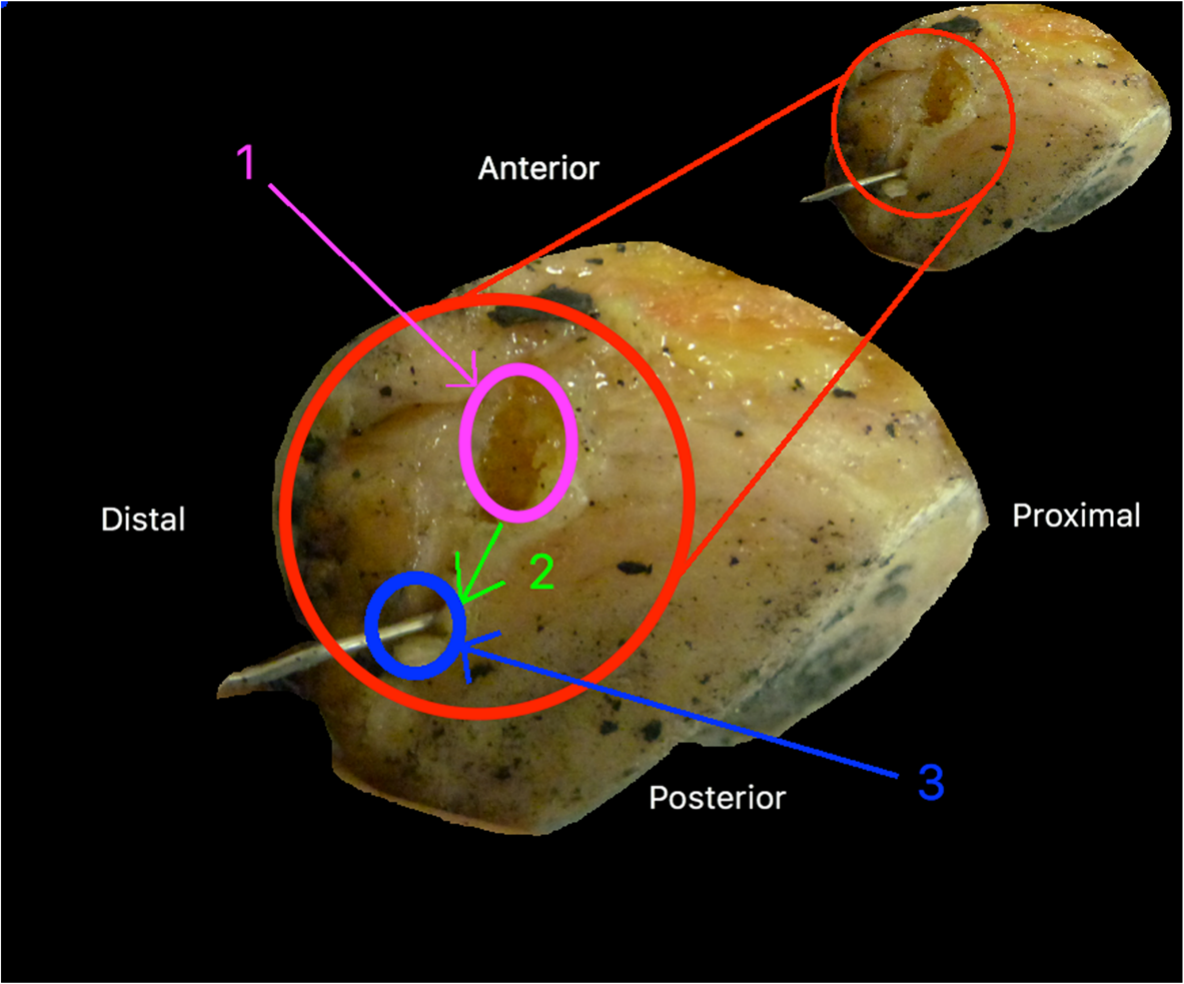
*« Cheese-wiring » effect of the steel frame at the level of the patella: 1: Orifice of the initial tunnel; 2: direction of the bone rail; 3: Orifice of the tunnel after “cheese-wiring” effect. A bone rail at both the patellar and the tibial levels was visible in the metal frame group and caused relaxation of the frame when returning to knee extension and therefore a loss of protection of ligament suture*

Steel wire caused a bony rail at the level of the patellar (Fig. 4) and tibial tunnels (7 of 12 knees), indeed it generated a “cheese-wiring” effect.

## Discussion

The study was conducted on strictly identical groups through distribution of an anatomical subject into two groups and a single technique of reinsertion. Measurement by an optical method involves no contact and is therefore non-disruptive and more accurate than an in vivo study. The establishment of mounting on a hinged knee prosthesis requires a single flexion with the involvement of the patella in the femoral trochlea.

Study on anatomical subjects does not take into account the post-lesional hematoma, per operative bleeding, exposure difficulties that may affect surgery, and it depends on the primary stability of the experimental set-up. The main limits of the anatomical model involve the properties of the chain extenders with no contraction/muscle shortening and partial dehydration of tendons and ligaments. The mean age of 69.3 years is debatable, since rupture of the patellar ligament regularly occurs in young patients (under 40 years) (Boggione & Marmorat 2004; Siwek & Rao 1981), most of them participants in athletics, a factor that may influence tendon elasticity (Kaux & Crielaard 2014) and bone strength. In vivo, the presence of quadriceps and more hydrated tendon would change the pre-tension angle of the frame, but not the protection that it provides for the suture. The setup done in this study allowed not to take these biases into account, as we were testing not the property of the tendon and its suture (tensile test), but rather the protective role of the frame for a flexion delta.

Comparably to the series of Kasten et al. (2001), protection of the suture was similar in the metal frame and the terylene frame. The results of this study did not find significant differences regarding delta flexion (*p* = 0.114) for disunity to 2 mm. Maximal admissible flexion delta didn’t differ between the steel and the terylene frames.

The main advantage of terylene is to avoid a surgical reintervention and the risks it entails of infection, pain and stiffness of the knee. The steel frame also causes tolerance problems, spontaneous rupture and lowering of the patella (Roudet et al. 2015). The choice of terylene was motivated by data from the literature: it is a polyester such as Dacron used in the series of Levy et al. (1987) and Mersilene in those of Lindy et al. (1995) and Miskew et al. (1980). In the series of Kasten et al (2001), the frame was in PDS cord but a tendency to infection was reported. The important thing is not the yarn brand but its polyester composition witch good biocompatibility (Li et al. 2006; Quester et al. 2002) and a conservation of its biomechanical properties (Privalova et al. 1988) in vivo.

It was noticed that unlike the metal wire, which didn’t show significant deformation, the terylene wire was deformed by lengthening during bending. Knee examination after the dynamic tests showed that the elongation of terylene is due not only to its elasticity but also to a progressive retightening of its knot. Slippage at the knot level can be explained by the properties of waxed terylene polyester, which tends to favor sliding. To limit this phenomenon, a domino system could replace the node during frame pre-tension.

Rigidity of the steel wire may explain the presence of tibial or patellar bone rails. The series of Roudet et al. (2015) also revealed an enlargement of the patellar tunnel of the steel frame in 1 out of 3 cases. On 7 out of 12 knees, a bone rail at both the patellar and the tibial levels was visible in the metal frame group and 0 in the terylene group. This “cheese-wiring” effect (Fig. 4) caused relaxation of the frame when returning to knee extension and therefore a loss of protection of ligament suture. The elasticity of the Terylene wire helps to avoid this problem. The tendon stretching allowed by the elastic properties of terylene ensures a better quality of ligament remodelling as shown by the study of Fujie et al. (2000) on rabbits.

The results showed that a ligament disunity of 1 mm was reached for a very low delta flexion. This means that when we exceed the flexion at which the frame has been stretched, the edges of the ligament suture quickly lose contact. It can be concluded that in practice, acceptable immediate postoperative flexion to avoid disunity and thereby promote healing should be limited to the flexion at which the frame has been stretched. The maximum angle of flexion in re-education could be reported on the prescription of physiotherapy and adapted to each case according to the flexion of intraoperative pre-tension of the frame. In this case, one might wonder whether the frame is useful, but currently all biomechanical studies show a better strength of the suture when it is reinforced by a frame that is, in metal (Shelbourne et al. 2001) or non-absorbable wire (Ravalin et al. 2002) or hamstrings (Mihalko et al. 2010). In fact, the frame serves as a flexion stop. Although the flexion that puts the frame in tension (flexion stop) may be exceeded by the strength of the leg flexor, the patient feels this stop and therefore encounters no more flexion.

An interesting alternative currently used by many centers and in particular presented in Roudet et al. study (Roudet et al. 2015) is the protection frame with a tendon from the crow’s feet (gracilis or semitendinosus). Mihalko et al. showed in a cadaveric study that this frame is stronger than a metal frame (Mihalko et al. 2010), and also avoids the removal of the material secondarily. However, it is difficult to produce for dancers, gymnasts and other athletes using hamstrings.

## Conclusions

The protection of a patellar ligament suture with a terylene wire seems to be an excellent alternative to the metal frame, which is the gold standard. There is no significant difference in terms of suture protection. The absence of reintervention for removal of material, discomfort of material and the « cheese-wiring » phenomenon makes this technique attractive compared to the gold standard. A randomized comparative in vivo study would be interesting to corroborate this work.

## Additional files

Additional file 1: Groups of patients. (XLSX 13 kb)

Additional file 2: Complete results of flexion angles, deformation percentage of frames and calculation of delta flexion angles for inter-suture gap of 1, 2 and 3 mm. (XLSX 37 kb)

## Abbreviations

% def: Deformation percentages
G_steel_: Group protected by a steel frame
G_terylene_: Group protected by a Terylene frame
mΔF: Mean delta flexion angle
ΔF: Mean delta flexion
